# Biomimetic Lattice Structures Design and Manufacturing for High Stress, Deformation, and Energy Absorption Performance

**DOI:** 10.3390/biomimetics10070458

**Published:** 2025-07-12

**Authors:** Víctor Tuninetti, Sunny Narayan, Ignacio Ríos, Brahim Menacer, Rodrigo Valle, Moaz Al-lehaibi, Muhammad Usman Kaisan, Joseph Samuel, Angelo Oñate, Gonzalo Pincheira, Anne Mertens, Laurent Duchêne, César Garrido

**Affiliations:** 1 Department of Mechanical Engineering, Universidad de La Frontera, Temuco 4811230, Chile; 2 Department of Mechanics and Advanced Materials, Campus Monterrey, School of Engineering and Sciences, Tecnológico de Monterrey, Av. Eugenio Garza Sada 2501 Sur, Tecnológico, Monterrey 64849, Mexico; s.narayan@tec.mx; 3 Master Program in Engineering Sciences, Faculty of Engineering, Universidad de La Frontera, Temuco 4811230, Chile; i.rios02@ufromail.cl; 4 Laboratoire des Systèmes Complexe (LSC), Ecole Supérieure en Génie Electrique et Energétique ESGEE Oran, Chemin Vicinal N9, Oran 31000, Algeria; menacer_brahim@esgee-oran.dz; 5 Facultad de Arquitectura, Construcción y Medio Ambiente, Universidad Autónoma de Chile, Talca 3460000, Chile; rodrigo.valle@uautonoma.cl; 6 Mechanical Engineering Department, College of Engineering and Architecture, Umm Al-Qura University, P.O. Box 5555, Makkah 24382, Saudi Arabia; molehaibi@uqu.edu.sa; 7 Industrial Finishing and Sustainable Manufacturing Lab, Department of Mechanical Engineering, Ahmadu Bello University, Zaria 810107, Nigeria; engineer.kaisan@gmail.com; 8 Department of Mechanical Engineering, Baze University, Abuja 900108, Nigeria; samuel.joseph@bazeuniversity.edu.ng; 9 Department of Materials Engineering (DIMAT), Faculty of Engineering, Universidad de Concepción, Concepción 4070138, Chile; aonates@udec.cl; 10 Department of Industrial Technologies, Faculty of Engineering, University of Talca, Camino a Los Niches Km 1, Curicó 3344158, Chile; gpincheira@utalca.cl; 11 Metallic Materials Science (MMS), A&M Department, University of Liège, 4000 Liège, Belgium; anne.mertens@uliege.be; 12 Department ArGEnCo-MSM, University of Liège, 4000 Liège, Belgium; l.duchene@uliege.be; 13 Department of Mechanical Engineering, Universidad del Bío-Bío, Concepción 4081112, Chile; cgarrido@ubiobio.cl

**Keywords:** lattice structures, additive manufacturing, mechanical optimization, energy absorption, computational modeling, biomimetic materials, topology optimization, smart materials, hierarchical lattices, multi-scale experimental validation

## Abstract

Lattice structures emerged as a revolutionary class of materials with significant applications in aerospace, biomedical engineering, and mechanical design due to their exceptional strength-to-weight ratio, energy absorption properties, and structural efficiency. This review systematically examines recent advancements in lattice structures, with a focus on their classification, mechanical behavior, and optimization methodologies. Stress distribution, deformation capacity, energy absorption, and computational modeling challenges are critically analyzed, highlighting the impact of manufacturing defects on structural integrity. The review explores the latest progress in hybrid additive manufacturing, hierarchical lattice structures, modeling and simulation, and smart adaptive materials, emphasizing their potential for self-healing and real-time monitoring applications. Furthermore, key research gaps are identified, including the need for improved predictive computational models using artificial intelligence, scalable manufacturing techniques, and multi-functional lattice systems integrating thermal, acoustic, and impact resistance properties. Future directions emphasize cost-effective material development, sustainability considerations, and enhanced experimental validation across multiple length scales. This work provides a comprehensive foundation for future research aimed at optimizing biomimetic lattice structures for enhanced mechanical performance, scalability, and industrial applicability.

## 1. Introduction

**Figure 1:**
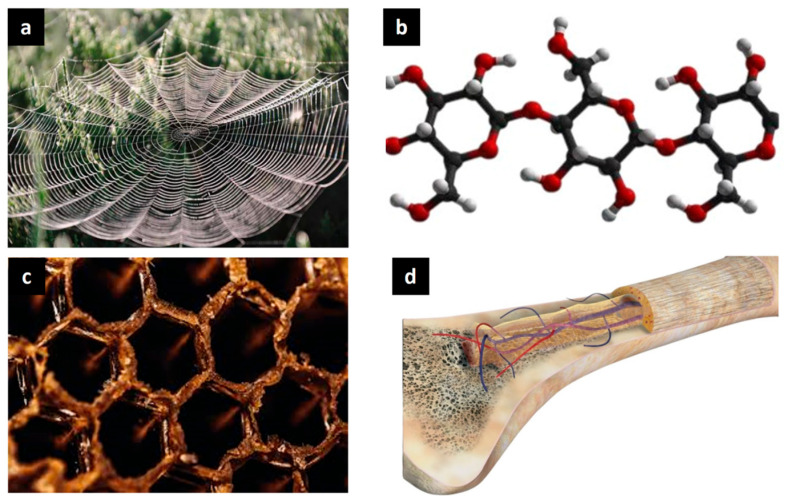
Naturally occurring lattice structures: (**a**) spider web [8], (**b**) cellulose structure, (**c**) honeycomb structure, and (**d**) human bone.

Cellular materials, encompassing foams, honeycombs, and lattices, are characterized by their porous formations. Lattice structures, a subset of cellular materials, are characterized by interconnected elements—struts, beams, surfaces, or complex geometries—arranged in a periodic grid-like pattern within a 1D, 2D, or 3D spatial configuration. These organized networks comprise repeating unit cells whose shape, size, and arrangement dictate the structure’s overall properties. Visualizations include a chain of repeating segments (1D), a planar grid such as a square, rectangular, or triangular mesh (2D), or a complex 3D network (e.g., cubic, tetrahedral, octet-truss structures, and triply periodic minimal surfaces). The periodic and organized nature of these interconnected elements imbues lattices with unique mechanical properties, including high strength-to-weight ratios, efficient load distribution, and tailorable energy absorption capabilities [1,2,3,4,5,6]. These structures are not a novel human invention; they are extensively found in nature (Figure 1). The rich repertoire of lattice structures evolved in nature to fulfill specific functions. For instance, spider webs (Figure 1a) exhibit exceptional tensile strength and energy absorption capabilities, enabling prey capture while withstanding environmental stresses. Cellulose networks (Figure 1b) form the structural foundation of plant cell walls, providing rigidity and resilience to plants [7]. Honeycomb structures observed in beehives (Figure 1c) demonstrate optimized space utilization and material efficiency, representing a highly efficient design. Human bones (Figure 1d) exhibit a trabecular lattice structure that combines strength and flexibility for weight-bearing and impact resistance. By studying these natural examples, researchers have been inspired to replicate similar architectures in synthetic materials. These bio-inspired lattice structures are applied in diverse fields, including aerospace, biomedicine, and automotive, where weight reduction without compromising structural integrity is a priority. Understanding the principles behind naturally occurring lattice structures allows engineers to optimize designs for enhanced mechanical properties, energy absorption, and functionality.

Cellular structures found several applications in fields of design, aerospace, automotive, and other engineering purposes due to their structural integrity and light weight [9,10,11,12]. The structure can be either a closed-cell or an open-cell type. In a closed-cell structure, solid plates separate the pores from each other, whereas in an open-cell structure, the pores are interconnected. Cellular structures can be classified into foams, honeycombs, and lattices. Cellular structures such as foams are often used as protective materials due to their high energy absorption capacity [13,14]. For instance, an impact shock test was developed to investigate properties of polyethylene (PE) foams to predict the failure modes and final fracture [14]. The graphite foam has high thermal conductivity and low density, so its use has been proposed for heat exchangers used for vehicle cooling application [15]. Flexible piezoelectric sensors based on honeycomb graphene macro-films were used to detect human movements [16]. Lattice frame materials such as octet, tetrakaidecahedron, face diagonal-cube, and cube were studied for turbine edge cooling applications [17]. Face diagonal-cube lattice structures demonstrated higher heat transfer coefficients and pressure drop values compared to cube lattices, despite cube structures exhibiting superior overall thermo-hydraulic performance due to lower pumping power requirements [17]. Recently, it was found that a bio-inspired robotic system builds high-strength lattice structures significantly more efficiently than additive manufacturing, achieving up to 131% greater compression strength and 180% higher elastic modulus [18].

Compared to conventional materials, lattice structures offer numerous advantages, including lower density, lighter weight, higher stiffness and strength, enhanced energy absorption, and superior acoustic insulation [19]. These properties make them well-suited for applications in aerospace [20], automotive [21], ship making [22], medicine [23], architectural design [24], and biomedical engineering [25].

**Figure 2:**
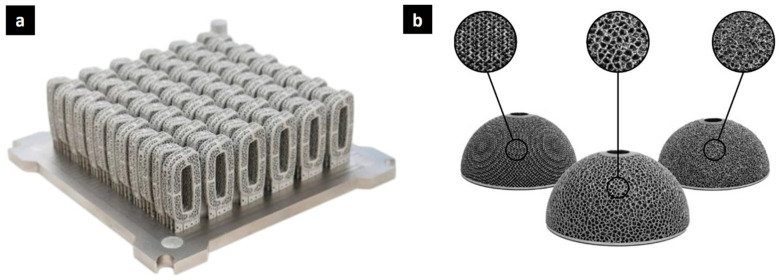
Spinal implants fabricated via electron beam melting: (**a**) single-layer build of a Ti6Al4V structure; (**b**) stochastic, trabecular, and engineered lattice structures.

In the biomedical field, lattice structures find applications particularly in the design of orthopedic implants as shown in Figure 2. Traditional solid implants, such as hip or knee replacements, can cause stress shielding, where the stiffness of the implant causes the surrounding bone to weaken over time. Lattice implants, on the other hand, can be designed to match the porosity and stiffness of natural bone, promoting better integration and reducing the risk of implant failure.

Figure 2a depicts the foundational layer of a spinal implant created through electron beam melting, highlighting the precise additive manufacturing process. Ti6Al4V, a titanium alloy, is commonly used for orthopedic implants due to its biocompatibility, excellent mechanical properties, including high strength [26] and low modulus [27], and corrosion resistance [28]. Visualizing the single-layer build emphasizes the controlled deposition of the material, which allows for complex geometries and customized designs.

Intricate lattice structures achievable with electron beam melting (EBM), including stochastic, trabecular, and engineered designs, are shown in Figure 2b. Stochastic lattices have a random pore distribution, offering potential advantages in mimicking the natural irregularities of bone. Trabecular lattices, inspired by the porous structure of cancellous bone, aim to replicate its interconnected network for enhanced bone ingrowth and mechanical stability. Engineered lattices, such as the octet-truss lattice [29], provide precise control over pore size, shape, and distribution, enabling optimization for specific mechanical and biological properties. These diverse lattice designs offer a range of mechanical properties and porosities, allowing for customization to match the specific requirements of the implant site and patient needs. The intricate designs achievable with EBM facilitate the creation of implants that promote bone ingrowth and reduce stress shielding [30].

In the aerospace industry, weight reduction is a constant pursuit. Every gram of excess weight in an aircraft or spacecraft translates to increased fuel consumption and reduced payload capacity. Lattice structures offer a promising solution to this challenge, thanks to their high strength-to-weight ratio and stiffness. By replacing solid components with optimized porous structures, aerospace engineers can create parts that are significantly lighter without sacrificing structural integrity. At the University of La Frontera, researchers developed a new type of optimization to create lightweight porous material that is 2.5 times stronger than traditional non optimized porous materials [31]. At MIT, researchers reported a lightweight lattice structure that is 10 times stiffer than traditional lightweight foams or honeycombs [32]. The optimized technique combined with the stiffer lightweight lattice structure from carbon fiber-reinforced polymers could be combined to create a new class of ultralight aircraft components such as fuselage panels, wind turbine blades, or satellite structures. In addition, topology-optimized lattice structures inspired by cuttlefish bone demonstrated to outperform conventional materials [33]. Therefore, research could explore the synergistic effect of the different reported strategies including biomimetic design strategies of lattice structures [33], the proposed optimization from Rilling et al. [31], to enhance the reported lightweight lattice structures in advanced carbon fiber composites structures [32] to improve stiffness and mechanical properties for high-stress applications.

**Figure 3:**
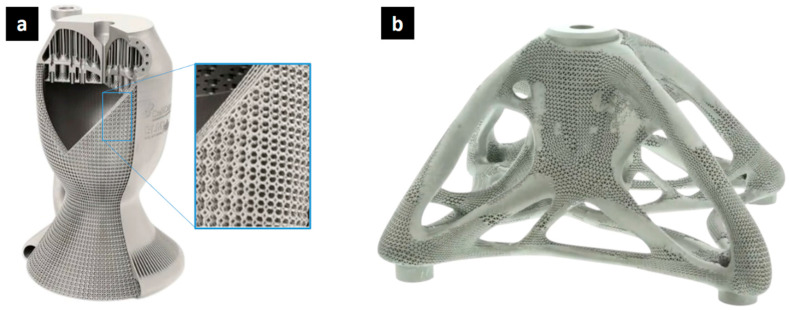
Applications of lattice structures in engineering: (**a**) One-piece rocket engine manufactured by CellCore GmbH and Nikon SLM Solutions using laser powder bed fusion with lattice structures within the engine walls to reduce weight and maximize surface area for efficient heat exchange. (**b**) A 3D-printed titanium spider bracket developed by Materialise, Altair, and Renishaw with a bio-inspired hybrid lattice structure for light weight and the strong connection of architectural glass panels.

Some successfully created parts demonstrate the advantages of lattice structures. For example, in the rocket engine in Figure 3a (a one-piece rocket propulsion engine from CellCore GmbH, Berlin, Germany, and Nikon SLM Solutions, Lübeck, Germany), weight was reduced by replacing solid walls with lattice structures. This not only decreases the overall mass, which is crucial in aerospace applications, but also improves performance by maximizing the heat exchange surface area. Products such as heat exchangers and engine blocks benefit from both the light weight and increased surface area provided by lattice structures. The metal lattice structure, fabricated by laser powder bed fusion, demonstrates the intricate geometries that can be achieved with additive manufacturing. Another example is the bracket in Figure 3b, a spider-shaped bracket designed by Materialise, Leuven, Belgium, in collaboration with Altair, Troy, MI, USA, and Renishaw, Wotton-under-Edge, UK. This titanium bracket, with a hybrid lattice structure inspired by biological structures, connects the corners of architectural glass panels, offering a lightweight yet strong solution with desirable thermal behavior. The biologically inspired design optimizes the use of materials while maintaining structural integrity.

A porous material energy-absorbing structure based on the ground of a biomimetic spider web structure was designed [34]. Reducing the cross-sectional diameter of the spiral resulted in a decrease in energy absorption. The honeycomb structures were applied to design of biomimetic bone scaffold [35]. The elastic modulus of the scaffolds matched with that of the elastic modulus of human cancellous bone, but the yield strength was much higher than that of the femoral neck [35]. An optimized model concerning both heat transfer and mechanical performances was presented to design the LS cooling channel with a variable aspect ratio in gas turbine blades [36]. The novel design had better heat transfer features, load-bearing capacity, and lightweight performances. Elastically isotropic plate and tube hybrid lattice structure was designed for a sound isolation panel [37]. Higher sound insulation up to 32 dB was found that was attributed to Helmholtz resonance and Bragg’s scattering. The development of biomimetic sound-absorbing components was developed using laser-sintered pine/phenolic resin composites, inspired by wood’s porous structure [38]. Results demonstrate that microstructural porosity enhances broadband sound absorption, particularly in mid-high frequencies with optimized performance through structural tuning and cavity design.

**Figure 4:**
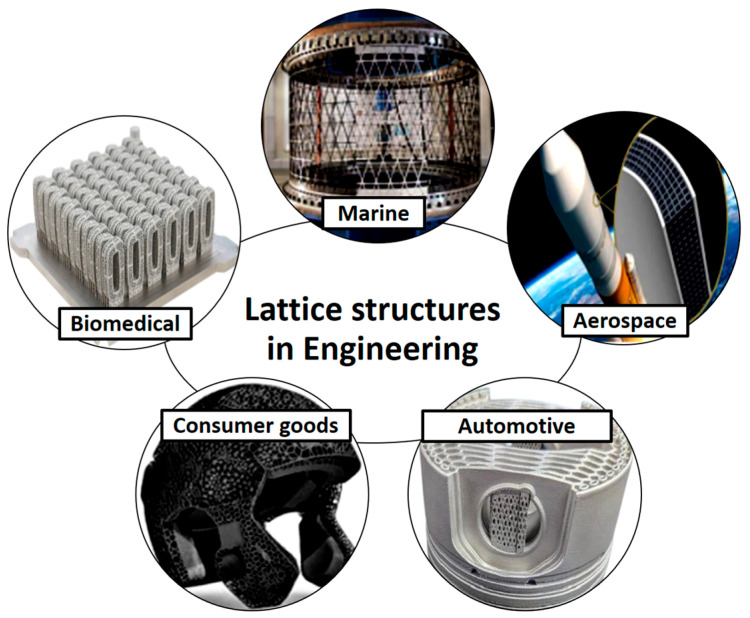
Applications of lattice structures.

Figure 4 presents a summary of the applications of these lattice structures in industries such as aerospace, automotive, and biomedical engineering. In lightweight aerospace components, they are used to reduce fuel consumption without compromising safety or performance. In automotive design, they are applied in crash absorbers and structural reinforcements to enhance passenger safety while minimizing vehicle weight. Biomedical applications include implants and scaffolds, where lattice structures promote bone in-growth and ensure biocompatibility.

The aim of this review is to provide a comprehensive analysis of lattice structures, focusing on their classification, properties, and applications. Drawing inspiration from natural designs, it explores how bio-inspired approaches could address critical engineering challenges. The scope also includes evaluating features such as load-bearing capacity, deformation, energy absorption, and the influence of manufacturing defects on performance. The review highlights advancements in additive manufacturing, hierarchical lattice designs, and smart materials to enhance structural functionality and adaptability. Key research gaps addressed in biomimetic design of lattice structures include predictive modeling, optimization of manufacturing processes, multi-scale experimental validation, and the integration of adaptive materials for achieving cost-effective and scalable material design innovations.

## 2. Current Design, Classification, and Manufacturing Methods of Lattice Structures

**Figure 5:**
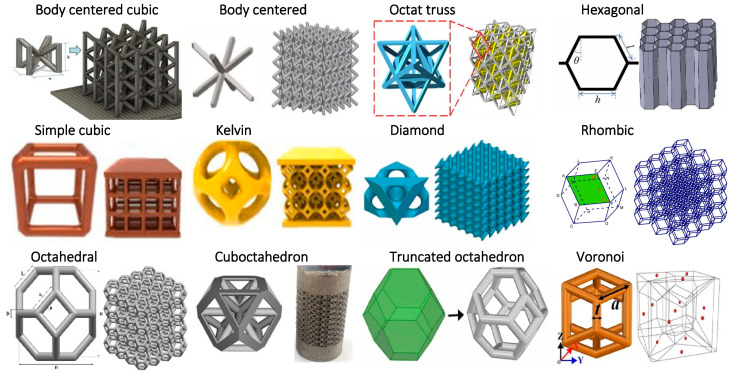
Shapes for strut-based unit cells: Vertically reinforced body centered cubic [39], Body centered cubic [40], Octat truss [41], Hexagonal [42], Simple cubic [43], Kelvin [43], Diamond [44], Rhombic [45], Octahedral [46], Cuboctahedron [47], Truncated octahedron [48], and Voronoi [49].

The properties of lattice structures depend on the geometry of cells, densities, sizes, dimensions, and arrangements. Based on the topology, they can be classified as the strut-based or surface-based cells. Strut-based lattice structures are comprised of struts and nodes. Figure 5 summarizes strut-based unit cells for lattice generation, encompassing both bio-inspired and engineered structures. Bio-inspired structure such as Voronoi, Kelvin cell, diamond, rhombic, and octahedral structures mimic the organization of biological systems and natural formations. On the other hand, engineered structures, such as the body-centered cubic, simple cubic, octet-trapezoidal, hexagonal, cuboctahedron, and truncated octahedron structures, prioritize mechanical optimization. It is important to note that some structures such as the diamond lattice can be considered both bio-inspired (found in crystallography) and engineered for structural applications.

Among the various strut-based unit cell shapes shown in Figure 5, body-centered cubic (BCC) lattice structures are widely studied due to their ease of fabrication. Extensive research focused on improving their mechanical properties and optimizing their designs. A hybrid lattice structure combining face-centered cubic (FCC) and BCC unit cells has been shown to increase strength by 74% compared to a pure BCC structure [50]. Additionally, design optimization of a T-BCC lattice structure has been performed to maximize stiffness [51]. Further advancements in BCC lattices explored structural modifications to enhance mechanical performance. A newly improved BCC lattice structure has been developed to improve load-bearing capacity by reducing stress concentrations [52]. Comparative studies of various BCC topologies demonstrated that a BCCZ structure exhibits a 62% increase in stress-carrying capacity [53]. Additionally, tapered strut designs in BCC structures have been investigated to mitigate stress concentrations at the nodes [54]. Recent advancements in computational methods also contributed to lattice design improvements. A deep learning-based approach has been used to tailor mechanical properties, optimizing design for enhanced stiffness, strength, and isotropy [55]. The behavior of additively manufactured Ti6Al4V BCC lattice structures has been studied, showing that while heat treatment had no effect on mechanical properties, strut diameter significantly influenced strength, toughness, and failure modes [56]. Beyond BCC lattices, researchers explored alternative strut-based designs to improve energy absorption and mechanical efficiency. A modified rhombic dodecahedron lattice structure with enhanced energy absorption and mechanical properties was developed by altering strut cross-sections, and its performance was validated using 3D-printed samples and finite element analysis (FEA) [57]. A parametric method for Voronoi-based lattice design was proposed, establishing a functional relationship between porosity, seed point density, and beam radius [58]. Additionally, a periodic array-based scaffold modeling approach was introduced, where the topological structure of Voronoi cells was controlled using polyhedron volume (V), face-centered scaling factor (F1), and body-centered scaling factor (F2). The mechanical properties of these scaffolds were quantified using compressive testing and simulations [59]. The influence of unit cell topology on the mechanical properties, printability, and permeability of lattice-inspired Voronoi-based metamaterials (LIVMs) has been further investigated, showing that thickness-graded Ti6Al4V Voronoi structures exhibit increased compressive strength with densification [60,61]. Additionally, studies on tapered beam designs in octet-truss and truncated octahedron unit cells revealed improvements in elastic modulus and reductions in elastic anisotropy [62]. The energy absorption capacity of the rhombic dodecahedron lattice was found to be higher than that of Kelvin and truncated cuboctahedron lattice structures based on selective laser melting (SLM) fabrication using AISI 316L material [63]. Lastly, the bending behavior of Ti6Al4V octet-truss lattice structures fabricated using EBM was studied to provide a simplified model to predict their mechanical performance [64].

**Figure 6:**
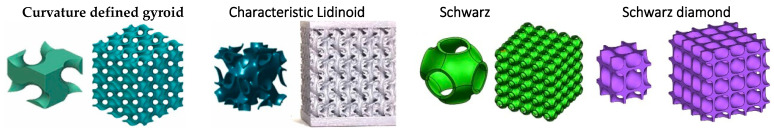
Shapes for surface-based bio-inspired unit cells: Curvature defined gyroid [44], Characteristic Lidinoid [66], Schwarz [67], and Schwarz diamond [68].

Surface-based lattice structures are generated from predefined surfaces or geometries, where the lattice is designed to follow surface contours and boundaries, optimizing material distribution and mechanical properties. These triply periodic minimal surfaces (TPMS) are considered bioinspired designs because they mimic naturally occurring geometries found in biological systems. These surfaces minimize material usage while maintaining structural efficiency, similar to biological membranes, trabecular bone, and cellular structures in living organisms. Mathematical equations, particularly partial differential equations, are commonly used to create minimal surfaces for lattices [65]. Trigonometric equations can be used to define the lattice pattern or layout, allowing the creation of complex structures, periodic or non-periodic, with precise control of their geometry and performance characteristics. Modifying the equation controls the shape, size, and density of the 3D structure. Figure 6 shows a summary of surface-based lattice shapes.

**Figure 7:**
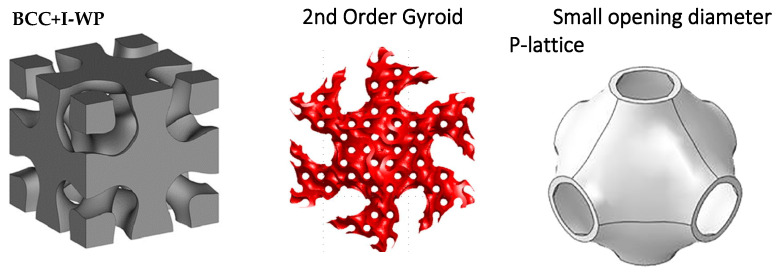
Geometry of modified TPMS lattices structures with precise control: BCC+I-WP [70], 2nd Order Gyroid [69], and Small opening diameter P-lattice [71].

Triply periodic minimal surfaces are a popular choice and are defined by mathematical functions that satisfy these equations. By adjusting parameters within these equations, such as cell size, shape, density, or other geometrical features such as strut thickness or surface smoothness, the final lattice structure can be precisely controlled [69,70,71] (Figure 7). Numerical methods and computer tools are often employed to approximate solutions and visualize complex TPMS structures, allowing for precise control and tailored designs.

The design of lattice structures is governed by application-specific requirements such as mechanical strength, energy absorption, heat dissipation, and biocompatibility. Computational tools such as FEA, topology optimization, and machine learning-based generative design enable the development of highly optimized lattice structures tailored for specific loads and environments. Advanced design software, including Autodesk Within, nTopology, Netfabb, Materialise Magics, Siemens NX, and Altair Inspire, have been instrumental in facilitating lattice design automation, allowing engineers to explore novel topologies that were previously unachievable.

The fabrication of lattice structures relies on various manufacturing techniques, which can be categorized as conventional subtractive methods, additive manufacturing (AM), hybrid manufacturing approaches, and alternative fabrication techniques. A comparison of these methods is presented in Table 1.

Conventional methods, such as machining, drilling, and etching, involve the removal of material from a solid block to create lattice structures [3,72]. While these methods allow high precision, they are limited in scalability and complexity, particularly for intricate internal geometries. Additionally, material waste is a significant drawback, making them less efficient compared to AM techniques. AM revolutionized lattice fabrication, enabling complex geometries through layer-by-layer deposition. Popular AM techniques for lattice structures include SLM and EBM, which are suitable for metallic lattices with high precision, but are prone to residual stresses and porosity [46]; fused deposition modeling (FDM), which is used for polymer-based lattice structures, offering flexibility but lower resolution; and stereolithography (SLA), which produces high-accuracy lattice structures mainly in polymeric materials. Despite its advantages, AM is prone to defects, such as porosity, surface irregularities, and anisotropic properties, which necessitate post-processing treatments such as heat treatment, hot isostatic pressing (HIP), and surface polishing. Hybrid AM integrates additive and subtractive manufacturing techniques to enhance structural accuracy and mechanical performance. For example, a lattice structure may be 3D printed and then machined for improved surface finish and dimensional accuracy. Hybrid AM combines the flexibility of AM with the precision of subtractive methods, but its complexity and cost remain higher than standalone techniques. Several alternative manufacturing approaches have been explored to address specific scalability, material diversity, and cost challenges associated with AM. Investment casting assisted by additive manufacturing involves AM to create a mold, which is then used for casting metallic lattices [73], offering advantages such as suitability for intricate geometries and better material flexibility than direct AM, but challenges include susceptibility to porosity, heterogeneity, and increased costs due to combined AM/casting processes. The direct foaming process introduces gas bubbles into a molten material to create lightweight, open-cell lattice structures [74], which is cost-effective and applicable to metals and ceramics, but challenges include difficult pore size control, heterogeneity, and defect formation due to inconsistent bubble dispersion. Wire-woven methods create strong, interconnected lattice structures from metallic wires [75], providing a high strength-to-weight ratio and good pore control but facing limited scalability for 3D complex geometries and low production efficiency. Powder metallurgy compacts and sinters metallic powder to form lattice structures [76], offering precise porosity control and material flexibility but being expensive for large-scale production and constrained in size due to sintering limitations. A study by Richard and Kwok [77] explored the rapid investment casting method for fabricating lattice topologies, including rhombohedral, Kelvin cell, cubic, and octet-truss structures, with findings indicating that lattices with higher node connections exhibited increased porosity and premature solidification, leading to compromised mechanical performance, whereas optimized element distribution improved mechanical integrity and reduced defects. Computational modeling played a crucial role in refining lattice manufacturing methods. A study by Chen et al. [46] systematically analyzed and optimized additive manufacturing parameters to improve mechanical performance. Machine learning algorithms have been applied to predict manufacturing defects, optimize process parameters, and enhance lattice designs for specific applications [78]. Advanced X-ray micro-computed tomography has been utilized to characterize macro- and meso-scale defects in AM-fabricated lattices [73], while optical and scanning electron microscopy provided insights into microstructural properties, allowing further optimization of AM techniques. Despite significant advancements, challenges remain in lattice structure manufacturing, particularly in terms of scalability, where AM-based lattice structures remain limited in large-scale production, defect mitigation requiring advanced process optimization to address porosity, residual stress, and dimensional inaccuracies, multi-material fabrication where future research should explore multi-material AM for functionally graded lattices, and computational-driven optimization where integrating AI and digital twin models could improve the predictive accuracy of lattice manufacturing processes. The evolution of lattice structure manufacturing has been driven by advances in additive manufacturing, hybrid approaches, and alternative fabrication techniques. While AM offers unparalleled design freedom, manufacturing defects and scalability challenges necessitate ongoing research into optimization strategies, computational design integration, and novel hybrid techniques. The future of lattice fabrication will likely rely on multi-scale modeling, AI-driven optimization, and high-precision manufacturing technologies to further enhance mechanical performance and expand practical applications in biomedical, aerospace, and structural engineering.

## 3. Manufacturing Defects in LPBF-Fabricated Lattice Structures: Causes, Measurement Techniques, and Mitigation Strategies

**Figure 8:**
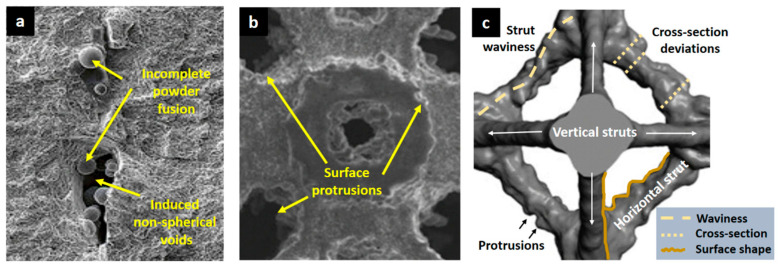
Main defects in lattice structures manufactured additively using LPBF: (**a**) Porosity due to incomplete powder fusion. (**b**) Surface protrusions and nodal distortion. (**c**) X-ray-reconstructed lattice cell showing several surface defects, and more a irregular shape for horizontal struts than vertical (build direction)-oriented struts (Adapted from Echeta et al. [79]).

Lattice structures produced through laser powder bed fusion (LPBF) additive manufacturing processes can induce several manufacturing defects depending on the complexity of their geometry and the limitations of the fabrication device. Defect formation in laser powder bed fusion is a significant concern, impacting the structural integrity and performance of fabricated parts, especially lattice structures. Several factors influence defect formation, including melt pool instabilities, heating/cooling dynamics, and inconsistencies in energy input. These factors can lead to three main defect types: porosity, residual stresses, and surface defects. Porosity arises from incomplete melt powder (Figure 8a) or trapped gases within the rapidly cooling melt pool. These gases may originate from voids in the initial powder bed or from the evaporation of low melting-point components. Existing ridges or protrusions (Figure 8b) on previously solidified layers can also trap gases and contribute to porosity formation. Incomplete melting, a subset of porosity defects, occurs when insufficient energy input prevents complete powder melting, resulting in irregular voids containing unmelted powder. This can affect multiple layers if the melt pool fails to penetrate and remelt the underlying material fully. In materials prone to oxidation, the formation of oxide layers further hinders wettability and exacerbates melt-related defects. Residual stresses develop due to the steep thermal gradients and rapid cooling rates inherent to the LPBF process. The rapid solidification of the melt pool leads to variations in thermal contraction across the part, generating internal stresses. Specifically, within lattice structures, LPBF defects manifest as dimensional inaccuracies, surface irregularities, and porosity (Figure 8c). Dimensional inaccuracies primarily stem from process-induced variations in strut diameters, nodal distortions, and orientation-dependent deviations within the lattice structure [79]. Surface irregularities can include roughness, balling, and the aforementioned protrusions, which can act as stress concentrators and reduce mechanical performance. Porosity, as discussed earlier, weakens the struts and compromises the overall structural integrity of the lattice.

Extensive research has shown that horizontal struts experience significant oversizing, often exceeding their designed dimensions, whereas vertical struts are prone to undersizing due to incomplete fusion and insufficient energy absorption [79]. Additionally, material accumulation at nodes leads to deviations from the intended geometry, which may negatively impact mechanical properties. Surface irregularities, including stair-stepping effects and surface protrusions, are exacerbated in down-skin surfaces due to the overhanging nature of the features, leading to localized overheating and poor heat dissipation. Down-skin surfaces exhibit significantly higher roughness than up-skin surfaces. This is due to weaker direct powder support, which, in turn, results in excessive heat build-up and interaction with loose powder. These factors contribute to increased material adhesion, melt pool instability, and uneven solidification. Porosity, the critical defect influencing mechanical integrity, occurs due to gas entrapment and insufficient melting, with its prevalence increasing in horizontal struts and nodal regions where thermal gradients are most extreme [79]. These defects collectively reduce mechanical strength, introduce stress concentrations, and negatively impact fatigue resistance.

The measurement techniques used to evaluate these defects include X-ray computed tomography (XCT), scanning electron microscopy (SEM), and optical microscopy. XCT has proven to be the most comprehensive tool for evaluating all three defect types, allowing for detailed analysis of dimensional deviations, surface topography, and internal porosity. However, XCT is limited by resolution constraints, particularly in detecting micro-porosity, and requires long acquisition times. Optical microscopy and SEM, while effective for analyzing surface features and strut morphologies, are constrained by the inability to penetrate internal lattice regions.

Strategies to minimize manufacturing defects include optimized design constraints, process parameter tuning, and post-processing techniques. Design optimizations such as adjusting strut diameters based on orientation-dependent deviations and incorporating functionally graded lattice structures have been shown to significantly reduce dimensional inaccuracies [79,80,81]. Process parameter adjustments, including optimizing laser power and scanning speed, help mitigate strut oversizing and surface defects, with controlled energy density for ensuring uniform material consolidation [82,83,84]. Post-processing techniques, particularly chemical etching [85,86] and hot isostatic pressing [87], could be effective in reducing surface irregularities and eliminating residual porosity.

Despite these advancements, research challenges remain in fully characterizing and predicting defect formation, particularly in complex geometries and multi-material lattice systems, highlighting the need for further investigation into in situ monitoring and adaptive process controls.

## 4. Performance Analysis of Lattice Structures

Stress-based performance evaluation of lattice structures involves several key steps, beginning with an investigation into the optimized stiffness and strength of the lattice design. This process includes optimization to address scale separation and size effects, ensuring that mechanical properties remain consistent across different structural scales. Advanced generative design techniques, such as multi-agent systems (MAS) [88], FEA [4,89], and response surface methods (RSM) [90], are commonly employed to enhance the design methodology. Additionally, adaptive modifications based on non-uniform stress distribution further refine the structural integrity, while surrogate models, such as decision tree algorithms, are utilized to predict the stress–strain response with improved accuracy.

Metallic lattice structures are renowned for their exceptional energy absorption capabilities, making them ideal for aerospace and transportation applications. Recent studies delved into various design strategies to enhance these properties. Wang et al. [91] introduced a hierarchical circular-cell lattice structure, demonstrating that such configurations significantly improve mechanical properties and energy absorption capacity. Their findings suggest that incorporating hierarchical designs can lead to superior performance under compressive loads. Similarly, Top et al. [92] investigated functionally graded lattice structures (FGLSs) and found that varying the density and orientation of unit cells within the lattice enhances mechanical performance. This gradation allows for tailored energy absorption characteristics, which are crucial for impact mitigation in dynamic environments. In another study, hybrid lattice structures combining different unit cell geometries were explored to overcome the limitations of single-lattice designs. The research demonstrated that these hybrid configurations exhibit superior energy absorption under compressive loads, highlighting their potential for applications requiring high impact resistance [93]. Furthermore, the design of hierarchical architected lattices has been shown to enhance energy absorption efficiency. By introducing multiple levels of hierarchy within the lattice structure, a four to fivefold increase in energy absorption was achieved under low loads and strain rates, showing promising results for high-speed and energy impact applications [94]. Recently, a bio-inspired curved-elliptical (BCE) lattice structure was introduced to enhance the mechanical performance and deformation stability of lightweight three-dimensional lattice architectures, addressing the inherent trade-off between strength and stiffness [95]. BCE lattice specimens were fabricated using SLM and subjected to quasi-static compression tests, complemented by FE simulations for validation. The results demonstrate that BCE lattices exhibit a bending-dominated delocalized deformation mode, preventing catastrophic collapse and enabling controlled energy dissipation. By optimizing design parameters such as the number of peaks (N) and curve amplitude (A), the BCE lattice structure achieved a specific energy absorption (SEA) of 24.6 J/g at a relative density of 8%, marking a 48.5% improvement over the octet lattice and a 297.6% increase compared to the BCC lattice. Additionally, its crushing force efficiency (CFE) surpassed those of the octet and BCC lattices by 34.9% and 15.8%, respectively [95]. N refers to the quantity of wave-like curvatures along a single strut of the lattice unit cell, with a higher N generally enhancing deformation control and mechanical stability. Parametric studies confirmed that adjusting N and A significantly influences the compression deformation mode, stiffness, and fracture resistance, integrating the advantages of tensile-dominated and bending-dominated lattice structures. These findings demonstrate the potential of bio-inspired BCE lattice structures for aerospace, automotive, and impact-absorbing applications, offering optimized mechanical performance, lightweight design, and structural stability through SLM-based fabrication and numerical optimization. Despite this progress, a full characterization and defect-free or defect formation predictions, particularly in complex geometries and multi-material lattice systems, are still not fully reached. Ongoing research should focus on in situ monitoring and adaptive process controls to ensure manufacturing consistency and structural integrity of innovative design and manufacturing approaches that optimize and enhance the energy absorption properties of metallic lattice structures for critical applications.

To analyze and tailor the performance of lattice structure under deformation loads, computational and numerical analysis is generally applied. The deformation patterns can be classified into quasi-static, transition, and dynamic modes [96]. The deformation modes obtained with simulated computer models show that a local “I”-shaped deformation zone improves the energy absorption capacity of lattice. Zhang et al. simulated pomelo peel-inspired hierarchical honeycomb to show that the SEA of the hierarchical honeycomb under out-of-plane and in-plane compression conditions was 1.5 times more than the traditional honeycomb, and the equivalent platform stress was 2.5 times higher than that of the traditional structure [97]. Lu et al. [98] simulated hierarchical chiral structures to show that plateau stress and energy absorption efficiency of the anti-tetrachiral structure were higher than those of the hexachiral structure. Further numerical studies by Habib et al. [99] with nonlinear FEA analyzed crushing response and energy absorption characteristics. The tensile buckling-dominated structure had strong stiffness and strength, but lower energy absorption performance. The simulations of Lin et al. [100] proved that the triangular hierarchical honeycomb performed and absorbed at least twice the energy. Pyramidal lattice structures of three strut materials prepared by 3D printing combined with investment casting and direct metal additive manufacturing eliminated stress fluctuations in plateau stages [101].

Machine learning (ML) models have been increasingly used to predict SEA and mean crushing force (MCF) in polycrystalline-like lattice structures made of different steels [102]. ANSYS 2023 R1 finite element modeling considering elastic–plastic and the hardening behavior of the materials and geometrical non-linearity was used to study the energy absorption and stiffness in various lattice structures [103,104,105]. Surface quality and geometric discrepancy of NiTi lattice struts made by laser powder bed fusion were studied, with a focus on fatigue-related stress concentration factors [106]. The results show that strut diameters and inclination angles played a significant role in geometry inaccuracy, surface texture, and stress concentration factor distribution on the surface.

A simulation-driven design tool was developed to design metal lattices with a target compressive stress–strain curve. The design tool was applied to five distinct curves and validated via manufacturing and testing of an optimized design. The unit cell aspect ratio and strut taper design variables improved strength and energy absorption in lattices [107]. Node stress concentration is a key factor affecting the mechanical performance of lattice structures [108]. An equal-strength body-centered cubic (ES-BCC) lattice structure was additively manufactured using 316L stainless steel via SLM. The results of a mechanical compression test and FEA reveal that the failure location of the ES-BCC structure changed from the nodes to the center of the struts. At the same density, the energy absorption, elastic modulus, and yield strength of the ES-BCC structure increased by 11.89%, 61.80%, and 53.72% compared to the BCC structure, respectively [108].

**Figure 9:**
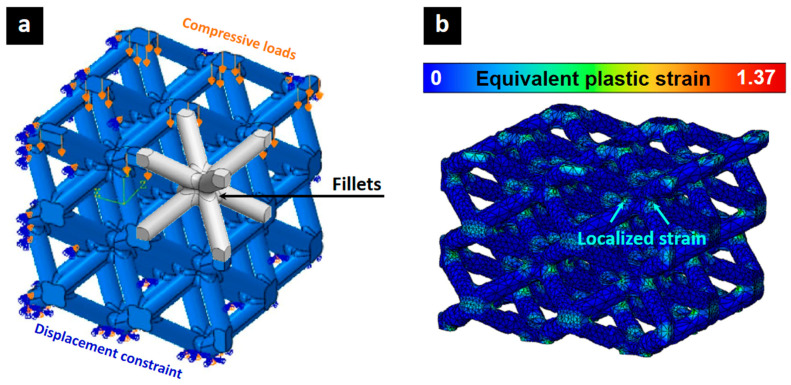
(**a**) Applied fillets in the lattice model to reduce stress concentration. (**b**) Compressed lattice structure showing plastic strain occurring locally at node intersections of struts [111].

Dara et al. [109] reveal that stress concentration at nodes has a significant impact on the stability of plateau stress, deformation modes, and energy absorption capacity in lattice structures. The study emphasizes various strategies to mitigate the effects of stress concentration, including optimizing lattice topology, utilizing graded structures, and employing post-processing techniques. By reducing stress localization at nodes, these approaches improve the structural integrity of lattice structures, delay the onset of densification, and enhance impact resistance. In particular, fillets at strut intersections are an effective way to distribute stress more evenly (Figure 9), reducing failure risks and improving load-carrying capacity [109,110,111]. Optimizing node geometry with graded fillets while taking into account creep and buckling constraints further improves structural performance, minimizing stress concentrations and increasing overall durability.

Residual stresses developed during AM can influence the mechanical performance of structural components. In particular, AM induces residual stresses in lattice structures, affecting their mechanical performance [112]. These stresses come from thermal gradients and material phase changes during the printing process. Residual stresses affect deformation behavior in lattice structures, producing warping, bar curvature, and node deviation, particularly in low-density and BCC topologies. In the case of SLA printing, the stresses are primarily caused by polymerization shrinkage and differences in resin solidification rates [112]. Several experimental techniques can be used to quantify the effects of residual stresses. Digital image correlation (DIC) provides strain measurements during testing, enabling the identification of stress-induced distortions. XRD and Raman spectroscopy can detect internal stresses in polymer-based structures. Thermomechanical analysis (TMA) evaluates the expansion/contraction behavior to understand that shrinkage-induced stresses and FEA with inherent deformation methods can correlate experimental results with simulation-based stress predictions. By integrating these methods, the optimization of lattice structure design and manufacturing processes can be achieved.

FE simulations for plate and cube-shape geometries in convergence were compared to experimental residual stress results available in the literature [113]. Results show that lattice geometry affects the distribution and magnitude of residual stresses. The lattice samples made by 3D printing were observed experimentally and validated numerically by using FEA [114]. Experimental data were compared with those obtained from ANSYS material designer simulations for their linear elasticity. Then, compressive strength and stiffness were taken from the experimental data. Remarkably, among all three specimens, octet structure had a good dynamic behavior and tended to resonate at much higher frequencies than the other samples. Additionally, in terms of compressive strength, cubic structure had the highest value [114]. Gradient hollow-strut octet lattice structures produced through LPBF were also studied for their mechanical behavior, particularly in relation to geometrical parameter effects on yield strength, Young’s modulus, and energy absorption [115]. These parameters were then optimized using FEA to enhance structural efficiency. DIC experimental technique in addition to FEM were investigated in 316 L stainless steel lattice structures of different geometries manufactured by laser-based PBF and subjected to shear loading [116]. The stiffness of the lattice structure under shear does not follow the Maxwell’s equations. BCE lattice specimens with different parameters were fabricated using SLM technology [95]. FE numerical simulations demonstrate that the proposed BCE lattice structures exhibited stronger mechanical performance and more stable deformation modes that can be adjusted through parameter tuning [95]. An arc-shaped strut lattice structure made from thermoplastic polyurethane (TPU) material using fused deposition modeling process was tested under uniaxial compression [117]. The energy absorption parameters were compared with other TPU structures in the reported literature.

**Figure 10:**
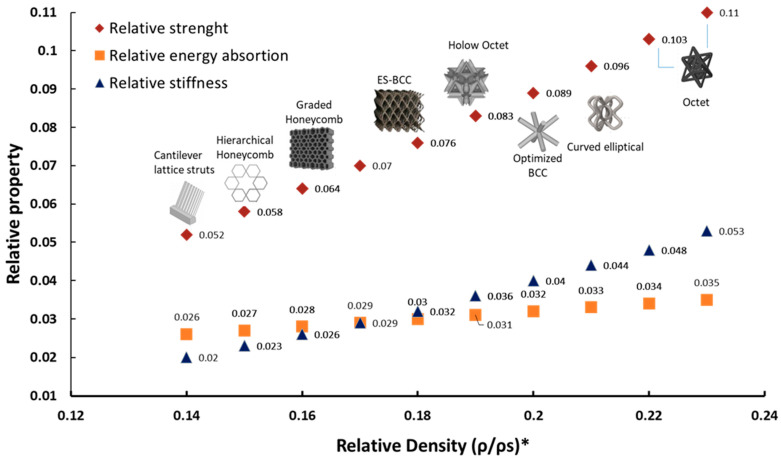
Increasing trend of relative properties of lattices with increasing relative density.

To evaluate the ability of the lattice structures to withstand compressive loads before failure, the compressive strength of the lattice (*σ*∗) is computed. It is influenced by factors such as geometry, material properties, and relative density. In contrast, the strength of the solid material (*σ*_*s*_) refers to the compressive strength of the bulk material from which the lattice is made, without considering its porous or cellular architecture. The ratio *σ*∗/*σs* is a key parameter in assessing the mechanical efficiency of lattice structures, helping to determine how much strength is retained compared to the solid material while benefiting from weight reduction and material savings. Table 2 summarizes the relative compressive strength (*σ*∗/*σ*_*s*_) and specific energy absorption of lattice structures according to Gibson–Ashby equations. The SEA of lattice structures, measured in MJ/kg, was computed using the Gibson–Ashby framework, which relates lattice material properties to their relative density. SEA is defined as the energy absorbed per unit mass and is calculated using the relationship *SEA* = (*σ*∗/*ρ*∗) = (*σs*/*ρs* )·(*ρ*∗/*ρs*) 0.5, where *σ*∗ is the compressive strength of the lattice, *ρ*∗ is the density of the lattice, *σs* is the compressive strength of the bulk material, and *ρs* is the density of the bulk material. The ratio *ρ*∗/*ρ*s represents the relative density of the lattice, which directly influences its mechanical behavior. In this study, stainless steel (316L) was selected as the reference bulk material, with an assumed compressive strength of 200 MPa and density of 8000 kg/m^3^. The Gibson–Ashby model suggests an exponent of 0.5 for stretch-dominated lattices, indicating that SEA scales with the square root of relative density. The computed SEA values reveal that higher-density lattices tend to absorb more energy, which is crucial for applications in crashworthiness, impact protection, and lightweight structural optimization (Figure 10).

A clear trend is observed where higher relative density lattices exhibit greater strength, stiffness, and energy absorption. For example, the octet-truss lattice [104] with a relative density of 0.23 achieves the highest relative strength (0.110), energy absorption (0.056), and stiffness (0.053), emphasizing its superior mechanical efficiency for load-bearing applications. Conversely, low-density lattices, such as the NiTi lattice struts [106] with a relative density of 0.14, exhibit the lowest values for these parameters, highlighting the trade-off between mass reduction and mechanical integrity. Considering lattice structures categorized as being bending-dominated (e.g., honeycombs, pyramidal, and polymeric lattices) and stretch-dominated (e.g., octet, BCC, and graded lattice structures), the data of Table 2 confirm that stretch-dominated lattices, such as the octet-truss and equal-strength BCC, outperform bending-dominated counterparts in terms of strength and stiffness, as they distribute loads more efficiently along their structural elements. For instance, the equal-strength BCC lattice [108] exhibits a relative strength of 0.076, surpassing that of traditional honeycomb structures (0.058–0.070), which rely on bending deformation. These results validate the superior load-bearing potential of stretch-dominated designs, particularly in high-performance applications such as aerospace and crashworthiness engineering.

A critical consideration for lattice structures is their energy absorption capacity, particularly in impact-resistant and crash energy management applications. The functionally graded lattice [103] and the arc-shaped strut lattice (Patel and Pandey, 2024 [117]) demonstrate improved SEA while maintaining moderate stiffness, making them suitable for shock-absorbing applications. Table 2 also highlights that hierarchical designs, such as the gradient hollow-strut octet lattice [115], improve both energy absorption and structural stability, reinforcing the advantages of multi-scale architectures.

The trade-off analysis in lattice design for engineering applications shown in Figure 8 allows for summarizing the comparison of the different analyzed lattice shapes in terms of strength, stiffness, and energy absorption. While high-density stretch-dominated lattices provide excellent mechanical performance, they may not always be ideal for applications requiring extreme mass reduction. In contrast, low-density honeycomb-based lattices offer lightweight solutions with moderate mechanical properties, making them preferable for applications where energy dissipation and impact resilience are prioritized over absolute strength. The performance trends observed in the Table 2 align with theoretical predictions from the Gibson–Ashby model and reinforce the importance of lattice topology and relative density optimization. The results suggest that stretch-dominated lattices should be prioritized for applications demanding high strength and stiffness-to-weight ratios, while hierarchical and functionally graded lattices are more suitable for impact and energy absorption scenarios. These findings provide a framework for optimizing lattice structures in aerospace, biomedical, and automotive industries, where a balance between weight savings, mechanical performance, and energy dissipation is crucial. These studies highlight the critical role of numerical and experimental validation in advancing lattice structure design. Ongoing research continues to refine fabrication techniques, optimize energy absorption, and address residual stress concerns for more reliable and efficient lattice-based applications in various engineering fields.

## 5. Optimization Strategies for Lattice Structures: Computational Methods, Bio-Inspired Approaches, and Advanced Manufacturing Integration

Optimizing lattice structures for high performance involves tailoring their geometric parameters (cell size, shape, and topology) and material properties to meet specific application requirements. This often means maximizing properties such as strength-to-weight ratio, stiffness, energy absorption, or thermal conductivity, while minimizing weight and material usage. Early work on lattice structures focused on simpler geometries, such as honeycombs and foams. Analytical methods and empirical formulas were used to estimate their mechanical properties. The lack of advanced computational tools limited the complexity of designs and the scope of optimization. However, this period established the fundamental understanding of the relationship between lattice geometry and performance. Currently, new optimization techniques integrated in computational tools such as FEA [118] allows for evaluating lattice behavior under various loading conditions.

**Figure 11:**
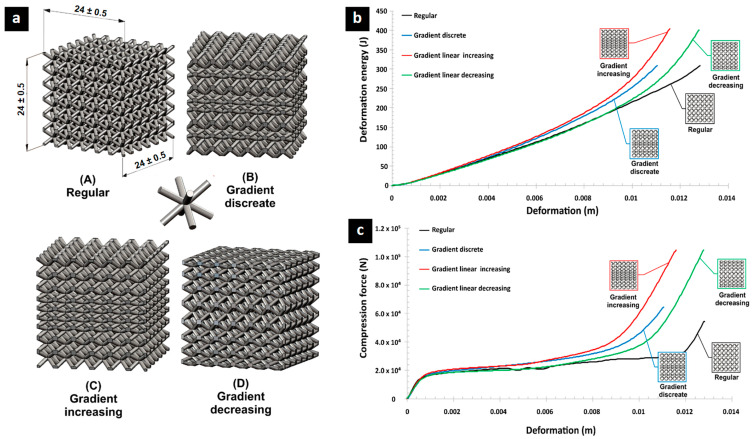
Compressive impact load response of different optimized lattice structures. (**a**) Developed and tested lattice structures. (**b**) Deformation energy and (**c**) compression force evolutions with deformation [119].

Previous research focused on optimizing the mechanical behavior or lattice specimens subjected to quasi-static and dynamic compression loading [119] using both regular and three different variants of SS 316L lattice structures with gradually changed topologies (discrete, increase, and decrease), and designed and additively manufactured with the use of the selective laser melting technique (Figure 11). The results show significant higher compressive strength and energy absorption performance compared to regular lattice structure. A gradient lattice structure has been designed based on multiscale topology optimization [120], constructing various types of volume parametric lattice. An anisotropic design and optimization method was also developed for conformal gradient lattice structures, with an adaptive orientation and variable period. These resulting anisotropic conformal lattice structures were much stiffer than uniform and directly mapped designs [120].

Topology optimization methods, including density-based and level-set approaches, enable the automatic generation of optimal lattice designs based on specified objectives and constraints [105,121]. Response surface optimization techniques [122] efficiently explore the design space to identify optimal parameter combinations combined with additive manufacturing such as laser powder bed fusion, and the fabrication of complex lattice structures and the realization of designs previously impossible to manufacture are achieved [123].

The integration of bio-inspired design principles led to the development of novel lattice structures with enhanced performance characteristics. Notable work Kladovasilakis et al. from [118] explored topology optimization of orthopedic hip implants using bioinspired lattice structures and FEA. Starting with the incompatibility of solid metal implants with bone tissue, the investigated Voronoi, Gyroid, and Schwarz Diamond lattices to reduce weight, increase porosity, and enhance mechanical efficiency showed that Schwarz Diamond topology demonstrated superior strength and minimal stress concentration under in vivo loads. With the integration of this bioinspired design and engineering optimization, the study successfully created lightweight, porous implants mimicking trabecular bone structure validated through FEA simulations for determining stress distribution, safety factors, and overall performance. The lattice structures are demonstrated to reduce material usage by up to 38%, while maintaining load-bearing capacity and the functional gradation further enhanced performance.

Research has been exploring machine learning (ML) algorithms to accelerate the optimization process and discover new high-performing lattice topologies. ML-based models were used to analyze the design variables effecting performance of lattice structures [124]. A machine learning-based approach accelerates the design and characterization of lattices by enabling accurate prediction of effective metamaterial properties through regression models such as random forest, while facilitating optimized microstructure design using the aquila optimizer. This expedites the discovery of microstructures with diverse and outstanding characteristics, significantly reducing simulation time and improving the efficiency of developing tailored metamaterials [125]. A neutral network has also been proposed for design of lattice metamaterials [126]. A generative approach was used to accelerate the optimization process [127]. Optimized structures showed stiffness improvement by 59%, strength increase by 49%, toughness improvement by 106%, and isotropy increase by 645%. The artificial intelligence process needed relatively few simulation calls [128]. A new lattice core for sandwich structures was designed using machine learning, producing unit cells with a significantly higher load capacity than traditional designs, such as the octet cell, achieving 261–308% higher buckling resistance and 13–35% greater flexural strength [129]. ML was used to print the shape memory polymer (SMP) metamaterials [130] and to fabricate spatially varying lattice structures [131]. A machine learning model trained on simulated data may provide fast and approximate predictions, but its reliability for experimental validation depends on the fidelity of the training data [26,132]. This is particularly relevant for lattice structures, where simulations that incorporate simplifications (e.g., inherent strain methods) or omit real-world manufacturing imperfections such as microstructural variations, thermal gradients, and process instabilities may lead to reduced predictive accuracy and limited generalizability [133]. To improve reliability, training should incorporate experimental data, uncertainty quantification, and domain adaptation techniques. Hybrid approaches combining FEM-generated and real-world data enhance robustness, while techniques such as transfer learning can refine predictions for new manufacturing conditions.

The future of lattice optimization lies in integrating advanced simulation techniques, artificial intelligence, and novel manufacturing processes. Multi-scale modeling will enable more accurate predictions of lattice behavior across different length scales. Machine learning will automate the design process for identifying the optimal cell designs and structures, and even discovering new lattice topologies. Advancements in additive manufacturing, such as 4D printing, will open up new possibilities for creating lattice structures with tailored functionalities and adaptive properties [134]. The integration of smart materials and embedded sensors will enable the creation of responsive and adaptive lattice structures.

**Figure 12:**
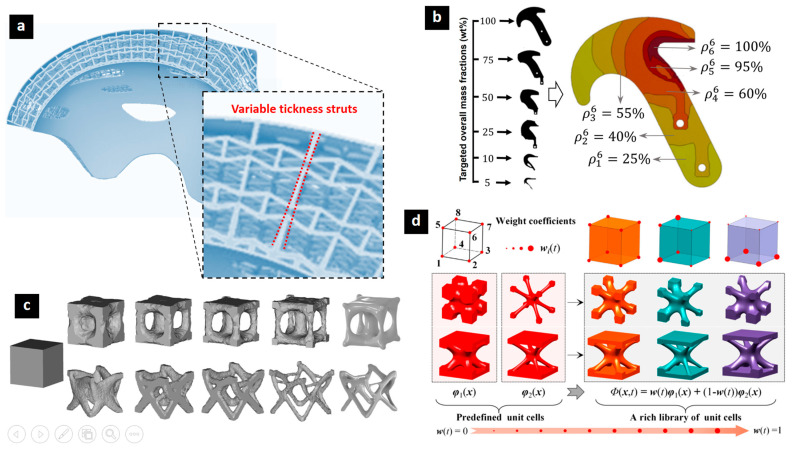
Density-based and level-set optimization approaches of lattice structures. (**a**) Optimized variable lattice thickness in helmet. (**b**) Iterative infill density (cell volume fraction) optimization [31]. (**c**) Iterative level-set optimization of two different lattices [139]. (**d**) Double-stage optimization of lattice structures using hybrid level-set-based predefined lattice cell and weight optimization at cell nodes [140]. Note that multiscale optimization can be reached by combining methods (**b**) with (**c**) or (**d**).

Continued research into optimizing lattice structures for specific applications, such as biomedical implants [135], lightweight aerospace components [118], and energy absorption systems [136], will further drive innovation in this field. Evolution in the field has to pass from simple analytical methods to advanced computational techniques integrated with the innovative additive manufacturing capabilities. Topology optimization technique, focusing on finding the optimal distribution of material within a given design space, aims to maximize performance while minimizing weight (Figure 12). The common approaches include the following: density-based methods, level-set methods, and evolutionary algorithms. Density-based methods represent the design space as a density field and iteratively optimize the density distribution [105]. Level-set methods use an implicit function to represent the boundaries of the structure and evolve the function to optimize the shape [121]. Evolutionary structural optimization gradually removes less stressed material while reinforcing highly stressed areas. Heuristic-based methods use rules of thumb and experience to guide the optimization process. Size and shape optimization focuses on optimizing the dimensions and shapes of individual lattice elements (struts, beams, etc.) or the overall lattice structure, parameterizing the geometry and using optimization algorithms to find the best parameter values [2,5,122]. Multi-scale optimization technique considers the behavior of the lattice structure at different length scales [137], from the microstructural level to the macroscopic component level, aiming to optimize the material properties and microstructure of the lattice material in addition to the overall geometry [138].

Bio-inspired optimization, the most fascinating method, draws inspiration from natural structures, such as honeycombs, trabecular bone, and plant stems, to design and optimize lattice structures. It involves mimicking the hierarchical organization and adaptive properties of natural materials [118]. To accelerate the optimization process and discover novel lattice designs, machine learning-based optimization is the emerging technique. A machine learning algorithm can also be used to predict the performance of different lattice configurations, guide the search for optimal designs, and even generate new design concepts [141]. However, the choice of optimization approach depends on the specific performance objectives, manufacturing constraints, and the level of detail required in the design. Other review articles adding valuable resources for summarizing the state-of-the-art in a particular optimization field are given here for the reader [121,142].

## 6. Challenges in the Design and Performance of Biomimetic Lattice Structures

One of the primary challenges in the design of biometric lattice structures is stress distribution and resistance. Unlike conventional engineered lattices, biometric structures often incorporate irregularities that can lead to localized stress concentrations. The key factors contributing to stress-related challenges include heterogeneous load distribution, material inhomogeneity, fatigue and failure mechanisms, and anisotropic properties [143]. Studies contrast the performance of regular lattices, such as octet or cubic cell designs, with natural-inspired configurations such as trabecular bone structures. Research findings suggest that stress hot spots tend to form in transition regions where geometries shift from one pattern to another. Although these regions offer localized reinforcement in biological systems, in engineered lattice structures, they can become sites for early failure.

Deformation behavior is another critical challenge in the application of biometric lattice designs. Natural lattice structures exhibit an intricate balance between flexibility and rigidity, but replicating this behavior in artificial systems remains complex. The main issues encountered include the transition between elastic and plastic regimes, geometry-induced constraints, scaling effects, and manufacturing-induced defects. Contrasting studies have shown that while biomimetic lattices such as those inspired by bone structures can exhibit improved compliance, they also display unpredictable deformation modes under high strain rates. Additionally, research into honeycomb-inspired and fractal-derived lattices indicates that deformation properties can vary significantly with small changes in geometric parameters [144].

Energy absorption is a crucial performance metric in applications such as crash structures, protective equipment, and medical implants. While natural lattices evolved to optimize energy absorption, engineered versions face the following challenges: complex collapse mechanisms, the interplay between material properties and lattice architecture, strain rate sensitivity, and the density versus performance trade-off. Studies contrasting bio-inspired and traditional engineering lattice structures reveal that certain natural patterns, such as the hierarchical cellular structures found in wood or trabecular bone, provide superior energy absorption per unit mass. However, implementing such structures in synthetic materials introduces fabrication and scalability challenges, limiting their practical applications. The unit cell topology can be simplified to adapt it to the available manufacturing process [145]. For example, in the work by Oliveira et al. [146], the design and manufacturing of sandwich panels is shown through two-dimensional structures inspired by hierarchical structures. In this way, to preserve their mechanical qualities, their original deformation mechanism is sought [147]. In particular, the deformation mechanism of auxetic structures is dominated by the bending of their re-entrant elements [148]. In this way, it is possible to design two-dimensional cells whose bending of their re-entrant elements maintains the same stiffness condition as a hierarchical structure [149]. The findings from Singh et al. [150] provide insight into the behavior of sandwich panels and can help engineers optimize their design for engineering applications. Studies have shown that the stiffness and energy absorption in sandwich panels are superior to those of hierarchical structures [151,152]. Focusing on these critical pathways allows for the reduction in geometrical complexity while retaining the necessary stiffness and strength [153].

FEA is frequently used to study lattice structures, but its effectiveness is hampered by computational constraints and convergence issues when dealing with highly complex biomimetic geometries. Biomimetic structures exhibit anisotropic properties [33], complicating predictive modeling. Additionally, additive manufacturing introduces defects such as porosity, micro-voids, and layer adhesion inconsistencies, altering mechanical properties. Considering defect distributions in lattice structures can improve accuracy in performance predictions.

Strut-based lattice structures consist of interconnected slender members forming a network of open cells, with common configurations including BCC, FCC, and octet-truss designs. These structures are known for their high stiffness-to-weight ratios and are widely utilized in applications requiring lightweight yet strong materials. However, the mechanical performance of strut-based lattices is highly sensitive to the dimensions and connectivity of the struts. Manufacturing imperfections, such as variations in strut thickness or incomplete bonding at nodes, can significantly degrade their mechanical properties. Zhang et al. [154] demonstrated using micro-CT analysis and an image-based finite cell method using a Lemaitre damage model that has defects such has porosity and dimensional inaccuracies in additively manufactured strut-based lattices, adversely affecting their compressive strength and stiffness.

Intentional porosity in lattice structures can be strategically controlled to improve performance, whereas unintentional voids in additively manufactured bulk materials act as stress concentrators and reduce mechanical stability. Optimized lattice designs allow for improved mechanical strength [115], tailored energy absorption [90], and controlled deformation under load. Unlike randomly distributed voids in bulk materials [155,156], which introduce unpredictable weaknesses, structured porosity in lattices ensures efficient stress redistribution and increased resistance to mechanical failure [31,49,104].

Surface-based lattice structures, in contrast, rely on continuous surfaces that partition space into distinct regions, often derived from TPMS. Examples include gyroid, diamond, and Schwarz-P surfaces. These designs offer smooth transitions and inherent structural stability, leading to superior mechanical performance in certain applications. Surface-based lattices exhibit unique stress distributions and volume fractions, contributing to higher energy absorption capacities and improved mechanical properties compared to their strut-based counterparts. Li et al. [157] explored various TPMS-based designs for bone tissue engineering and found that adjusting the size and volume fraction of these surfaces allowed for tuning stiffness and yield strength to match different bone types. The choice between strut-based and surface-based lattice structures depends on specific application requirements, including mechanical performance, manufacturability, and functional integration. Strut-based lattices are relatively straightforward to design and fabricate using additive manufacturing techniques. However, they are more susceptible to stress concentrations and manufacturing defects, which can compromise their structural integrity. Surface-based lattices, while offering smoother stress distributions and potentially better mechanical properties, present challenges in design complexity and fabrication precision. Advanced modeling and simulation tools are essential to accurately predict the behavior of these complex geometries under various loading conditions [4,8,118,158].

## 7. Open Challenges in Predictive Modeling, Additive Manufacturing, and Multi-Scale Validation of Biomimetic Lattice Structures

Several unresolved issues remain in the field of biometric lattice design, necessitating further research in the following areas: predictive modeling and simulation, optimization of additive manufacturing techniques, multi-scale experimental validation, integration of smart materials, and all associated scalability due to the infancy of the research area.

### 7.1. Predictive Modeling and Simulation

Despite significant progress on predictive modeling and simulation, current models face limitations that hinder their ability to accurately predict the complex behaviors of lattice structures under various conditions.

Biomimetic lattice structures often exhibit intricate geometries and heterogeneous material properties, making it challenging to predict their mechanical performance accurately. Traditional modeling approaches may not capture the nuanced interactions within these complex architectures. Recent studies explored the use of ML to address these challenges. For instance, researchers demonstrated the use of ML-based approaches to build and interpret surrogate models for mechanical performance, aiming to accelerate the design process of lattice structures [124].

In addition, AM processes used to fabricate the lattice structures can introduce defects and variability that can significantly affect mechanical properties. Understanding and predicting these influences are crucial for reliable design. For instance, multiscale experiments and predictive modeling research highlighted how AM processes impact the failure characteristics of lattice structures, emphasizing the need for advanced modeling techniques to account for these factors [159].

The design space for lattice structures is vast, encompassing numerous variables such as unit cell topology, relative density, and material selection. Exploring this high-dimensional space efficiently requires advanced predictive models. One strategy that has shown some progress is the integration of machine learning with traditional computational techniques [124] to optimize design variables and speed up the computational design process for lattices, allowing the exploration of millions of potential design options.

Surrogate models, a type of data-driven model, approximate the behavior of complex systems for faster analysis and optimization. Using these models could improve the efficiency of high-fidelity simulations of lattice structures in the iterative design processes. ML approaches and surrogate models have been shown to effectively predict mechanical properties and guide the design process of various lattice structures [160]. Incorporating data-driven methods into the predictive modeling can also increase the accuracy with large data sets to inform the model development [78].

Despite the benefits of surrogate models and data-driven approaches, more robust computational models are needed to accurately predict stress, deformation, and energy absorption in complex biomimetic lattices. Advancing predictive modeling and simulation for biomimetic lattice structures is imperative to overcome current limitations related to complex geometries, manufacturing-induced variability, expansive design spaces, computational demands, and the integration of data-driven methodologies. Focused research in these areas will lead to more accurate predictions of mechanical behavior, facilitating the development of optimized, reliable lattice structures for various demanding applications such as aerospace, automotive, and medical industries.

### 7.2. Optimization of Additive Manufacturing Techniques

The optimization of AM techniques for lattice structures is a critical area necessitating further research due to several persistent challenges that impact the mechanical performance and reliability of these structures. Despite advancements in AM, issues such as manufacturing defects, geometric inaccuracies, and limited understanding of process–structure–property relationships continue to hinder the full realization of lattice structures’ potential. One significant concern is the prevalence of defects inherent to AM processes, such as porosity, incomplete fusion, and residual stresses, which can compromise the mechanical integrity of lattice structures. Zhang et al. [154] highlighted that defects such as porosity and dimensional inaccuracies in additively manufactured strut-based lattices adversely affect their compressive strength and stiffness. The necessity for optimized manufacturing parameters to mitigate defect formation is clear. Geometric deviations from the intended design are another critical issue. Factors such as thermal distortions and layer-wise fabrication can lead to dimensional inaccuracies, affecting the performance of lattice structures. As discussed in previous sections, the demonstrated deviations in strut dimensions due to manufacturing processes could lead to significant discrepancies between designed and actual mechanical properties; therefore, a precise control and optimization of manufacturing parameters is required to ensure geometric fidelity. Furthermore, the complex relationship between process parameters and material properties in AM is not yet fully understood. Boursier Niutta et al. [161] emphasized that the interplay between factors such as laser power, scanning speed, and hatch spacing significantly influences the microstructure and, consequently, the mechanical properties of the fabricated lattice structures. This complexity, similarly encountered in other AM processes [82,162,163] necessitates comprehensive studies to develop optimized parameter sets tailored for specific lattice designs and materials.

Controversies also exist regarding the optimal design of lattice structures for AM. While some studies advocate for topology optimization to achieve lightweight yet strong structures, others point out the challenges in manufacturing the resulting complex geometries. The difficulties discussed by Top et al. [92] are in fabricating functionally graded lattice structures, particularly dimensional deviations in fine geometries. While design optimization strategies can lead to superior performance of lattice structures in general, the associated manufacturing issues, particularly for complex biomimetics design, often offset these benefits.

In conclusion, the optimization of additive manufacturing techniques for lattice structures remains a not fully developed research area. Addressing manufacturing defects, ensuring geometric accuracy, and deepening the understanding of process–structure–property relationships are clearly essential steps toward realizing the full potential of lattice structures and complex bio-inspired design for practical applications.

### 7.3. Multi-Scale Experimental Validation

More extensive physical testing is required to validate numerical simulations and refine design principles for biometric lattices. Multi-scale experimental validation is crucial for understanding the behavior of biomimetic lattice structures across different length scales, from the microstructural level to the macroscopic performance. Current research often relies heavily on computational models, which, while powerful, require empirical validation to ensure accuracy and reliability. The importance of the validation and demonstrating experimental corroboration prevents models from failing to capture critical failure mechanisms inherent in complex lattice geometries. However, comprehensive multi-scale experimental studies are scarce, primarily due to the challenges associated with fabricating and testing specimens at varying scales. This gap leads to a limited understanding of scale-dependent behaviors, such as the transition from elastic to plastic deformation and the influence of microstructural defects on overall mechanical performance. Future research should focus on developing standardized multi-scale testing methodologies, using in situ high-resolution SEM and X-ray microtesting and nanomechanical testing. Advanced manufacturing devices to produce test specimens that accurately represent the hierarchical nature of biomimetic lattices with reduced defects are also required. Such efforts will enhance the manufacturing quality of lattice structures, predictive capability of computational models, and inform the design of more resilient structures and critical parts.

### 7.4. Integration of Smart Materials and Scalability Weakness

The incorporation of shape-memory alloys [164,165], auxetic materials [5,89,166], and adaptive composites [2] could enhance the performance of biometric lattices in dynamic environments. Incorporating smart materials into biomimetic lattice structures offers the potential for adaptive and responsive systems capable of real-time performance optimization. Smart materials, such as shape memory alloys and piezoelectric composites, can provide functionalities such as self-healing, vibration damping, and adaptive stiffness. Despite these advantages, the integration of smart materials into lattice architectures remains underexplored. Demonstration of the potential of diagnostic capabilities of smart materials in detecting faults and cracks in structural health monitoring has shown progress [167]. However, challenges such as material compatibility, manufacturing complexities, and the need for reliable control mechanisms hindered widespread adoption [168]. Controversies also exist regarding the long-term durability and fatigue performance of smart materials within lattice structures, with some studies reporting degradation under cyclic loading conditions. The integration of piezoelectric materials into lattice structures can improve fatigue resistance by enabling self-healing mechanisms, which counteract damage accumulation under cyclic loading [167]. The piezoelectric effect allows these materials to generate electrical charges in response to mechanical stress, which can stimulate healing processes and delay crack propagation. However, activating self-healing mechanisms can introduce stress concentration points, potentially leading to new fatigue cracks. To mitigate this, ensuring uniform healing and material compatibility is essential. Advanced fabrication techniques, such as multi-material additive manufacturing, facilitate the seamless integration of piezoelectric components, minimizing the risk of stress concentration points. While piezoelectric-enabled self-healing shows promise for improving the fatigue life of lattice structures, further research is needed to optimize fabrication processes and control mechanisms. Future studies should focus on developing robust algorithms for self-healing activation and conducting long-term performance evaluations to ensure the durability and reliability of these adaptive systems.

Functionally graded lattices embedded with phase change materials (PCMs) present an opportunity for enhancing thermal insulation in aerospace applications. The integration of PCMs within metallic lattice structures allows for efficient thermal energy storage and temperature regulation, as the metallic framework enhances heat transfer to and from the PCM [169,170,171]. However, this approach introduces significant challenges in material compatibility and manufacturing precision. The overall thermal performance of PCM–lattice composites depends on the lattice topology, PCM distribution, and additive manufacturing tolerances [172]. Process-induced defects, such as dimensional inaccuracies and incomplete encapsulation of PCM, may lead to unintended thermal inconsistencies. Furthermore, existing semi-analytical models for these composites require extensive experimental validation to account for deviations in the manufactured structures [169]. Addressing these issues through advanced multi-material printing techniques and improved predictive modeling will be crucial for developing effective thermal management solutions in aerospace applications.

Concluding this section, the practical application of biomimetic lattice structures is often limited by challenges related to scalability and cost efficiency as it is a still-active field of research. While additive manufacturing enabled the production of complex lattice geometries, scaling these processes for large-scale applications remains challenging due to inherent fabrication time, cost, and manufacturing defects. Investigating cost-effective manufacturing approaches and scaling strategies will be crucial in transitioning biomimetic lattice structures from laboratory research to real-world applications.

## 8. Conclusions

This work presents a systematic review of lattice structures, analyzing their classifications, properties, and applications. Lattice structures have broad industrial relevance, particularly in aerospace, biomedical engineering, and mechanical design, due to their high strength-to-weight ratio, energy absorption capabilities, and structural efficiency. Firstly, various types of lattice structures were classified and compared. Secondly, the mechanical behavior of these structures, including their stress distribution, deformation capacity, and energy absorption potential, was reviewed. Poisson’s ratio and topology optimization of lattice structures emerged as major research areas, with significant focus on strut-based architectures.

The reviewed works indicated that the biometric design of lattice structures presents substantial challenges, particularly in the areas of stress concentration, deformation predictability, and energy absorption under impact conditions. Current computational models, while effective for preliminary analysis, often fail to capture real-world mechanical behavior accurately. Discrepancies arise due to unaccounted manufacturing defects, residual stresses, and microstructural inconsistencies inherent in additive manufacturing processes.

Despite these challenges, significant progress has been made in recent years. Advanced manufacturing techniques, such as hybrid additive manufacturing and functionally graded lattice designs, enhanced mechanical reliability and customization capabilities. Notable research demonstrated improvements in hierarchical lattice structures, which exhibit superior stiffness and energy absorption compared to traditional designs. Studies also identified promising trends in integrating reinforcement mechanisms, such as node reinforcement strategies and multi-scale topology optimization, to improve mechanical stability. Furthermore, the development of smart and adaptive lattice structures, incorporating piezoelectric and shape memory materials, demonstrated potential for self-healing, real-time monitoring, and tunable mechanical responses. These advancements suggest promising directions for the next generation of biomimetic lattice materials.

## 9. Future Research

Future research must address several key areas to fully unlock the potential of these structures. Firstly, the refinement of predictive computational models is essential. The integration of machine learning algorithms and AI-driven optimization techniques can significantly enhance the accuracy of lattice simulations by accounting for manufacturing defects, residual stresses, and multi-scale mechanical interactions. Secondly, improvements in manufacturing reliability remain a crucial focus. Reducing the high costs associated with advanced fabrication processes, minimizing variability in production quality, and developing scalable methods for mass production are necessary steps for broader industrial adoption. Thirdly, further investigation into novel material combinations and hybrid lattice structures can enhance mechanical performance while maintaining lightweight characteristics. This includes exploring high-entropy alloys, ceramic–metal composites, and bioinspired polymers.

The potential of multi-functional lattice structures also remains largely untapped. Future research should also focus on integrating thermal regulation, acoustic dampening, and impact resistance properties into a single lattice system. This could lead to revolutionary applications in space exploration, high-performance sports equipment, and biomedical implants. Additionally, the exploration of multi-scale experimental validation is critical for bridging the gap between theoretical predictions and real-world performance. Standardized testing protocols must be developed to assess mechanical reliability across different length scales. Finally, economic and environmental considerations must be addressed. The energy-intensive nature of additive manufacturing poses sustainability concerns, necessitating the development of more efficient production techniques. Cost-effective material sourcing and recycling strategies for lattice structures should also be explored to improve the commercial viability of these materials. In conclusion, biomimetic lattice structures hold immense potential for revolutionizing multiple engineering fields. While notable progress has been achieved, challenges remain in refining computational accuracy, manufacturing scalability, and material efficiency. Through targeted research in these domains, the next generation of lattice materials can be designed for superior mechanical performance, cost-effectiveness, and sustainability, leading to widespread real-world applications.

## Figures and Tables

**Table 1:** Manufacturing methods for lattice structures.

Manufacturing Method	Description	Advantages	Disadvantages
Conventional/subtractive	Material removal processes such as machining, drilling, and etching are used to create lattice structures from a solid block [3,72].	Suitable for specific lattice types, high precision achievable, and established processes.	Limited complexity, material waste, challenging for intricate internal features, and scalability issues.
Additive manufacturing	Layer-by-layer deposition of material to build 3D structures. Includes techniques such as SLM, EBM, FDM, and SLA [46].	Complex geometries achievable, minimal material waste, design flexibility, and rapid prototyping.	Limited material selection compared to conventional methods, potential for manufacturing defects (porosity, anisotropy), and post-processing may be required.
Hybrid additive manufacturing	Combines additive manufacturing with conventional techniques. For example, a lattice structure may be 3D printed and then machined for enhanced surface finish or precision.	Combines advantages of both methods, enables complex designs with enhanced functionalities, improved surface finish and precision.	More complex process, requires integration of different manufacturing systems, and higher cost compared to individual methods.
Alternative methods	Additive manufacturing-assisted investment casting: a fused filament fabricated pattern is infiltrated with plaster to create a mold [73].	Intricate geometries, design flexibility, wider material selection for patterns, reduced lead times and tooling costs, and improved accuracy and surface finish.	Potential issues include porosity and structural heterogeneity, size limits based on casting capabilities, and combined AM/casting costs.
	Direct foaming: Gas bubbles are introduced into a molten metal (or other material) to create a foamed structure. This can be achieved through various methods, including gas injection or the addition of blowing agents that decompose and release gas [74].	Complex shapes and open-cell structures, relatively low cost compared to some other lattice fabrication methods, and can be applied to a variety of materials.	Difficult pore control (size, distribution, heterogeneity), limited architectural control (vs. 3D printing), viscosity hindering bubble rise/creating defects, and potential casting defects (porosity).
	Wire-woven methods: metallic wires are woven into a lattice structure [75].	Complex geometries, good pore control, and potentially high-strength/stiffness (depending on material and weave).	Lower production efficiency (especially for complex 3D shapes), limited scalability, and difficulties with complex 3D architectures and material integration.
	Interlocking assembly: fibers or elements are interlocked to form a lattice [32].	Can be relatively simple and low-cost, depending on the materials and interlocking mechanism used. Suitable for large-scale structures.	Fine structures are unavailable; limited to simpler geometries.
	Powder metallurgy: metallic powder is mixed with binders, compacted, then sintered at high temperatures [76].	Complex shape creation, good porosity control, material flexibility, and reduced waste through near-net-shape manufacturing.	Expensive for large production runs; limited in size due to equipment constraints; some materials are difficult to process; and post-processing may be required for tolerances and surface finish.

**Table 2:** Performance of lattice structures with shape, strength, and energy absorption.

Study	Focus Area	Key Findings	Lattice Shape	Relative Density (ρ/ρs)*	Relative Strength (σ/σs)*	Corrected Relative Energy Absorption (E/Es)*	Relative Stiffness (E/Es)*
[96]	Deformation modes in lattice structures	Identified quasi-static, transition, and dynamic deformation modes	Hierarchical honeycomb	0.15	0.058	0.027	0.023
[97]	Hierarchical honeycomb for improved SEA	SEA and equivalent stress higher than traditional honeycomb	Pomelo peel-inspired honeycomb	0.20	0.089	0.032	0.040
[98]	Hierarchical chiral structures’ energy absorption	Anti-tetrachiral structure superior to hexachiral	Hierarchical chiral structure	0.18	0.076	0.030	0.032
[99]	Polymeric lattice structures for energy absorption	Crushing response and energy absorption characteristics analyzed	Polymeric lattice	0.22	0.103	0.034	0.048
[100]	Bio-inspired hierarchical honeycombs	Crashworthiness enhanced via hierarchical structure	Triangular hierarchical honeycomb	0.17	0.070	0.029	0.029
[101]	Compressive properties of pyramidal lattice	Preparation method affected stress fluctuations	Pyramidal lattice	0.19	0.083	0.031	0.036
[102]	ML predictions of SEA and MCF in lattice structures	SEA and MCF successfully predicted using ML	Polycrystalline-like lattice	0.21	0.096	0.033	0.044
[103]	Functionally graded lattice structures’ energy absorption	Elastic–plastic modeling and stiffness evaluation	Functionally graded soft-hard lattice	0.16	0.064	0.028	0.026
[104]	High-performance AM lattice structures	AM enhanced mechanical properties of lattice structures	Octet-truss lattice	0.23	0.110	0.035	0.053
[106]	Surface and stress analysis of NiTi lattice struts	Strut diameters and inclination angles influenced stress concentrations	NiTi lattice struts	0.14	0.052	0.026	0.020
[107]	Simulation-driven design of metal lattice structures	Simulation-driven unit cell optimization improved performance	Metal lattice with unit cell optimization	0.20	0.089	0.032	0.040
[108]	Equal-strength BCC lattice mechanical performance	Failure location shifted from nodes to strut center, improving performance	ES-BCC	0.18	0.076	0.030	0.032
[115]	Gradient hollow-strut octet lattice properties	Geometrical parameters optimized via FEA	Gradient hollow-strut octet lattice	0.19	0.083	0.031	0.036
[113]	Residual stress in AM lattice geometries	Lattice geometry significantly affects residual stress distribution	Plate and cube-shaped lattice	0.15	0.058	0.027	0.023
[114]	Ductility failure analysis in AM lattice structures	AM lattices showed good dynamic behavior and high frequency resonance	Octet lattice structure	0.22	0.103	0.034	0.048
[116]	Shear behavior of 316L AM lattice structures	Stiffness under shear loading did not follow Maxwell’s equations	BCC lattice under shear loading	0.16	0.064	0.028	0.026
[95]	Curved-elliptical lattice structures for stability	Curved-elliptical structures exhibited strong mechanical performance	Curved-elliptical lattice	0.21	0.096	0.033	0.044
[117]	Arc-shaped strut lattice deformation analysis	TPU arc-strut lattices optimized for energy absorption	Arc-shaped strut lattice	0.19	0.083	0.031	0.036
